# Isavuconazole Treatment for Invasive Fungal Infections in Pediatric Patients

**DOI:** 10.3390/ph15030375

**Published:** 2022-03-19

**Authors:** Philippe Zimmermann, Benoit Brethon, Julie Roupret-Serzec, Marion Caseris, Lauriane Goldwirt, André Baruchel, Marie de Tersant

**Affiliations:** 1 Department of Pharmacy, University Robert Debre Hospital, AP-HP, 75019 Paris, France; philippe.zimmermann6@gmail.com (P.Z.); julie.roupret-serzec@aphp.fr (J.R.-S.); 2 Department of Pediatric Hemato-Immunology, University Robert Debre Hospital, AP-HP, 75019 Paris, France; benoit.brethon@aphp.fr (B.B.); andre.baruchel@aphp.fr (A.B.); 3 Department of General Paediatrics, University Robert Debre Hospital, AP-HP, 75019 Paris, France; marion.caseris@aphp.fr; 4 Pharmacology Department, Saint-Louis Hospital, AP-HP, Université de Paris, 75010 Paris, France; lauriane.goldwirt@aphp.fr; 5 Faculté de Médecine, Sorbonne Paris Cité, UMRS976 Université Paris Diderot, 75010 Paris, France

**Keywords:** pediatric, isavuconazole, invasive fungal infection

## Abstract

This work’s objective was to evaluate the safety of isavuconazole (ISA) as a treatment or prophylaxis for invasive fungal infections (IFIs) in immunocompromised children. IFI was reported as proven or probable according to international definitions. Therapeutic drug monitoring was performed using mass tandem spectrometry to quantify trough plasma concentrations. Targeted ISA levels were 2–4 mg/L, as reported in adult series. Nine patients received ISA as a curative treatment, and six received ISA as prophylaxis. IFIs were proven in four cases and probable in five. The median ISA trough plasma concentration in curative use was 3.19 mg/L [0.88;5.00], and it was 2.94 mg/L [2.77;3.29] in the prophylactic use. The median durations of treatment were 81 days [15;276] and 95 days [15;253], respectively. Three patients had elevated aspartate aminotransferase and alanine aminotransferase, and three patients had elevated creatinine serum. The IFI response was satisfactory in all cases at day 90. No side effects were reported. No patients developed an IFI. Our data underline the safety of an ISA 100 mg dosing regimen in children of <30 kg, which we recommend in this fragile population. We suggest that ISA plasma levels are monitored 10 days after ISA initiation and then every two weeks, alongside guided therapeutic drug monitoring (TDM) administration.

## 1. Introduction

Invasive fungal infections (IFIs) are significant complications in children treated for hematologic malignancies, as well as in allogeneic hematologic stem cells transplantation (HSCT) recipients [1,2]. Treating IFIs is challenging in this population. Systemic antifungal agents against invasive aspergillosis and mucormycosis include triazole and polyenes, which have unpredictable pharmacokinetics and toxicity profiles [3]. Therapeutic drug monitoring (TDM) is needed to guide antifungal administration. Treatments of underlining hematologic malignancies can be jeopardized due to drug interaction between the antifungal and chemotherapy, urging the need for new antifungal therapies. Isavuconazole (ISA) is a triazole that was recently approved by the European Medicines Agency for the treatment of invasive aspergillosis and mucormycosis in adults [4], with fewer adverse drug-related effects [5]. However, its use is off-label in the pediatric population, and optimal dosing and monitoring remain unclear. Articles and case reports suggest the administration of adult dosing in children over 30 kg, and half the dosing for children under 30 kg [6,7,8,9]. In those reports, tolerance, safety, and therapeutic response were evaluated, mostly in adolescent patients [6,10,11]. This work’s objective was to assess the safety of ISA in the pediatric population. For patients with an IFI, we also evaluated the clinical response.

## 2. Results

Among 15 identified patients, 9 received ISA as a curative treatment, and 6 as prophylaxis. The median age was 9 years [2;11], and the median weight was 29 kg [12;37]. Five patients were treated for B-cell acute lymphoblastic leukemia (ALL) and two for T-cell ALL; two for Fanconi anemia, two for aplastic anemia, one for acute myeloblastic leukemia, one for anaplastic large cell lymphoma, one for Burkitt lymphoma, and one for myelodysplastic syndrome. Ten patients underwent HSCT. Of those nine patients who received ISA as a curative treatment, four patients had a proven IFI. *Aspergillus flavus* was identified from nasal septum biopsy in one patient, and *Mucormycosis* was isolated from sinus biopsy, lung biopsy, and blood cultures, respectively, in three patients. Five patients were considered to have a probable IFI: *Aspergillus fumigatus*, diagnosed based on positive serum βD glucan (*n* = 2), positive serum galactomannan aspergillus antigen (*n* = 4), bronchoalveolar lavage cytology (*n* = 5), and positive CT chest (*n* = 5). Patient characteristics are reported in Table 1. All patients with a proven IFI were previously treated with liposomal amphotericin B. Of those five patients with a probable IFI, four received voriconazole, and one received liposomal amphotericin B. Patients received curative ISA at a median time of 17 days after IFI diagnosis. All the patients who received ISA as prophylaxis had not previously been treated with antifungal drugs.

### 2.1. Safety in Curative Use

Seven patients weighing less than 30 kg received a loading dose of ISA of 100 mg every 8 h for six doses, and then a maintenance dose of 100 mg once daily. ISA was administered intravenously (IV) in five patients and orally in two, for whom the ISA drug capsule was opened. Two patients weighing more than 30 kg received a loading dose of ISA of 200 mg every 8 h for six doses, and then a maintenance dose of 200 mg once daily. In all cases, ISA was administered as second-line therapy. All patients with proven IFI received ISA as salvage therapy, alongside concomitant systemic antifungal agents, either caspofungin or liposomal amphotericin B. Among the patients with probable IFI, two switched to ISA because of the toxicities of first-line antifungal drugs, and three switched to prevent drug interactions with chemotherapy or cyclosporine. During ISA treatment, six patients resumed chemotherapy, and four patients underwent HSCT and were treated with cyclosporine for graft versus host disease prophylaxis.

The first median ISA trough concentration (C_trough_) was 2.77 mg/L [0.73;5.12], within a median time of 4 days after the initiation. The dosing regimen had to be adjusted in five patients, and more than once in three of them. The ISA doses were increased in all five patients by 20%, and the ISA plasma levels were measured within a median time of 4 days after the dose adjustment. The median duration of treatment was 81 days [15;276]. The median ISA C_trough_ was 3.19 mg/L [0.88;5.00]. All patients receiving IV ISA at initiation shifted to oral formulation during the maintenance period. In the case of the two patients who received opened ISA drug capsules, the first ISA C_trough_ was 1.6 mg/L and 1.79 mg/L, respectively, and the dosing regimen had to be adjusted once. Five patients received a co-administration of drugs metabolized through cytochrome P450 (CYP) 3A4 (CYP3A4), including lorlatinib, venetoclax, or cyclosporine. The median ISA C_trough_ was 3.19 mg/L [0.88;3.72]. Dosage adjustments were as numerous for patients who did not receive drugs metabolized through CYP3A4. No side effects were reported, nor were there any significant drug-drug interactions.

Elevation of aspartate aminotransferase (AST) and alanine aminotransferase (ALT) (Common Terminology Criteria Adverse Event (CTC AE) grade 3) occurred in three patients, with ISA levels >5 mg/L in one of them, and complete resolution occurred with ISA interruption for 20 days. Creatinine serum level was elevated (CTC AE grade 1) in three patients without ISA overdose or discontinuation. No skin, gastric, hematologic, or cardiac toxicities were reported.

### 2.2. Safety in Prophylaxis Use

Six patients received ISA as IFI prophylaxis after HSCT. Two patients weighed <30 Kg and received the 100 mg regimen dosing, and four patients were treated with the 200 mg regimen dosing. The median ISA C_trough_ was 2.94 mg/L [2.77;3.29], and the median duration of treatment was 95 days [15;253]. ISA C_trough_ data were missing for one patient. The dosing regimen had to be adjusted in one patient, decreased two times, and then increased once. All patients received a co-administration of drugs metabolized through CYP3A4, cyclosporine, for graft versus host disease prophylaxis. No side effects were reported, nor were significant drug–drug interaction, and no patients developed an IFI.

### 2.3. Clinical Response

On day 15, four of nine patients with an IFI had a stable response, and five of nine had a partial response to therapy. On day 30, only one patient had a stable response, seven of nine had a partial response, and one had a complete response. Finally, on day 90, three of nine patients had a partial response, and six of nine had a complete response. Two patients died from a refractory hematologic disease.

## 3. Discussion

Our work retrospectively reported on a cohort of 15 pediatric patients with hematologic malignancies treated with ISA in a single-center institution.

In our study, nine of fifteen patients <30 kg received the 100 mg dosing regimen. In this population, the mean ISA level at 4 days after initiation was 2.59 mg/L [0.73;5.12], which was similar to levels described in the adult population. However, dose adjustments were needed in four patients <30 kg in the curative use, as initial plasma levels were lower than reference values. We also observed higher levels than the reference values in two patients <30 kg who received ISA with co-administered drugs metabolized through cytochrome CYP 3A4. Lower ISA levels may be attributed to an early first ISA C_trough_, despite the ISA loading dose, considering the ISA’s prolonged half-life of 120 h. First ISA C_trough_ values were also lower in patients receiving ISA capsules. However, these lower levels may not be solely attributed to capsules, as IV and oral formulations of ISA display the same bioavailability. Higher levels may be attributed to the co-administered drugs metabolized trough cytochrome CYP 3A4. Therefore, the first ISA C_trough_ after initiation showed interpersonal variability, mostly suggesting under-dosing in younger patients, probably due to an increased clearance and shorter drug half-life [10]. These results underline the complexity of obtaining a balanced ISA C_trough_ within the reference values in this fragile population, in which we do not recommend an increased loading dose of ISA in the 100 mg regimen, considering the risk of ISA overdose. In our cohort, ISA adjustments were cautious, with 20% increases or decreases, and ISA C_trough_ values were regularly realized to ensure that ISA levels were within the recommended range. Our patients displayed numerous risks of unbalanced ISA plasma levels, among which included their weight being <30 kg and the use of co-administered drugs. Considering the prolonged ISA half-life, we recommend closely monitoring ISA plasma levels, with a first ISA C_trough_ 10 days after initiation, and then every 15 days during the maintenance period, in order to efficiently adapt ISA doses in this fragile population. Our findings aligned with prior reports [5,6,11,12] and highlight the need for guided-TDM therapy. Our results also showed the ability of our hospital pharmacists to personalize doses of ISA capsules, adapting them to patients <30 kg with guided-TDM therapy.

In the curative ISA use, three patients (33%) presented with AST and ALT elevation. However, they all received concomitant hepatotoxic chemotherapy, and only one had ISA levels >5 mg/L. Therefore, the elevation of transaminases may not be solely attributed to the hepatotoxic effect of antifungal triazole, but also to the cytotoxic effect of antineoplastic therapy. Creatinine serum levels were elevated in three patients; however, all received concomitant liposomal amphotericin B. Therefore, the elevation of creatinine serum levels may not be solely attributed to ISA.

In our study, no patients showed progressive fungal disease, and no deaths were related to a fungal disease. All patients with a proven IFI were treated with ISA as a salvage therapy. Patients with a probable IFI switched to ISA because of the toxicities of first-line antifungal drugs and to prevent drug interactions with chemotherapy. IFI response was satisfactory in all patients, with six patients showing a complete response and three showing a partial response at day 90. A balanced ISA dosage was obtained in each patient, with an ISA C_trough_ within the range of 2–4 mg/L. ISA prophylaxis was also satisfactory, but too few patients were evaluated to draw conclusions. Importantly, ISA treatment was prolonged in both curative and prophylaxis use, and there was no suspension of chemotherapy or immunosuppressive therapy. Moreover, no adverse effects or drug–drug interactions were noticed when ISA was co-administered with drugs metabolized through the cytochrome CYP 3A4, with either a curative or prophylaxis use.

Our results align with previous reports, where patients received ISA as a salvage therapy for a proven IFI and showed a complete regression of the infection, with few adverse events and similar ISA exposure levels within the recommended range of 2–4 mg/L, as shown in Table 2 [6,7,8,10,11].

The limitations of our study include the consideration of cases from only a single-center institution, a study design based on a retrospective work with a small-sized sample, and the inclusion of both curative and prophylactic uses of ISA. In addition, the evaluation of ISA efficacy is limited by the concomitant use of other antifungal drugs in six of nine patients, and the lack of historical controls.

## 4. Materials and Methods

This retrospective single-center study reported on all patients aged from 1 to 18 years and treated with ISA for more than 7 days in a pediatric hematology department from January 2018 to December 2020. Data collection included demographic data and hematologic malignancy type and status. IFI status was reported as proven or probable, according to recent international definitions [13]. The following IFI characteristics were collected: infection site, infectious agents, previous antifungal prophylaxis, oral or intravenous administration of ISA, and duration of treatment. When administered orally, the opening of the ISA drug capsule was documented. The co-administration of drugs metabolized through cytochrome P450 (CYP) 3A4 was also reported. TDM was performed using mass tandem spectrometry to quantify trough plasma concentrations. Targeted ISA levels were 2–4 mg/L, as reported in adult series [14,15]. Response to antifungal drugs was assessed 15, 30, and 90 days after the initiation of treatment and was defined as complete, partial, or progressive according to the European Organization for Research and Treatment of Cancer criteria [16]. The following safety laboratory data were collected: aspartate aminotransferase (AST), alanine aminotransferase (ALT), and serum creatinine at the initiation of ISA and peak value at any time. Transaminases were considered elevated if they were >3 times the upper limit of normal, and serum creatinine if >1.5 times increased from baseline. Adverse events were graded according to the National Cancer Institute’s Common Terminology Criteria (CTC AE) (version 5.0) [17]. Parents gave written informed consent for the retrospective analysis of clinical data, according to the Declaration of Helsinki principles.

## 5. Conclusions

In this retrospective analysis, our data underline the safety of an ISA 100 mg dosing regimen in children <30 kg. We recommend the 100 mg dosing regimen in this fragile population, and suggest that ISA plasma levels are closely monitored, leading to early and efficient dose adjustment. Adjustments of ISA doses should be cautious, with 20% increases or decreases. ISA plasma levels should be monitored 10 days after ISA initiation and when co-administered with drugs metabolized through cytochrome CYP 3A4, and then every two weeks during the maintenance period. The use of personalized doses of ISA capsules should also be considered. Further randomized and controlled studies are needed to confirm the dosing regimen and the efficacy of ISA in pediatric patients with hematologic malignancies.

## Figures and Tables

**Table 1:** Overview of patient characteristics.

Fungal Infection	Sex, Age (y), Weight (kg)	Hematologic Disease	ISA Initial DoseRoute	Co-Antifungal	Drugs Metabolized by CYP3A4	Time to First ISA Level (days)	First ISA C_trough_ (mg/L)	Capsules Opened at Any Time	Total Days of ISA	Median ISA C_trough_ (mg/L)	Modification of Dosage	Balanced Dosage mg/day	IFI Response at Day 15	IFI Response at Day 90	AST Spike (UI/L)	ALT Spike (UI/L)	Scr ^h^ Spike (µM)
Proven mucor ^a^	F 5.4 y 20 kg	ALCL ^c^	100 mg IV	L-AmB ^e^	lorlatinib	5	1.77	NO	105	2.14	Increased, 2 times	200	partial	complete	33	15	72
Proven mucor ^a^	M 6.5 y 19 kg	B-ALL	100 mg IV	L-AmB ^e^	NA	3	2.77	NO	64	2.58	Increased, 3 times	200	stable	partial	269	339	42
Proven mucor ^a^	M 2.4 y 13 kg	B-ALL	100 mg Oral	L-AmB ^e^	NA	9	1.6	YES	276	2.30	Increased, 1 time	130	stable	complete	34	22	55
Proven Asp ^b^ flavus	F 10.9 y 30 kg	T-ALL	100 mg IV	CASP ^f^	CsA ^g^	8	0.73	NO	356	3.22	Increased, 1 time	200	stable	complete	59	83	29
Probable Asp ^b^ fumigatus	F 2.4 y 12 kg	B-ALL	100 mg Oral	NA	CsA ^g^	7	1.79	YES	74	0.88	NA	100	partial	complete	59	35	22
Probable Asp ^b^ fumigatus	M 9.1 y 37 kg	Fanconi anemia	200 mg Oral	NA	CsA ^g^	5	3.87	NO	15	3.72	NA	200	partial	partial	98	79	56
Probable Asp ^b^ fumigatus	M 11.2 y 30 kg	Burkitt ALL	200 mg IV	NA	NA	4	3.29	NO	95	4.76	NA	200	stable	complete	399	394	46
Probable Asp ^b^ fumigatus	M 9.1 y 29 kg	B-ALL	100 mg Oral	NA	NA	5	4.2	NO	65	5.00	NA	100	partial	complete	258	234	35
Probable Asp ^b^ fumigatus	F 5.3 y 15 kg	MDS ^d^	100 mg IV	CASP ^f^	venetoclax/CsA ^g^	3	5.12	NO	81	3.19	Increased, 2 times	140	partial	partial	18	7	30
Prophylaxis	F 4.6 y 18 kg	AML	100 mg IV	NA	sorafenib/CsA ^g^	2	3.8	YES	240	2.77	NA	100	NA	NA	64	72	30
Prophylaxis	M 15.3 y 48 kg	Fanconi anemia	200 mg Oral	NA	CsA ^g^	NA	NA	NO	253	NA	NA	200	NA	NA	108	105	50
Prophylaxis	M 15.6 y 58 kg	T-ALL	200 mg Oral	NA	CsA ^g^	7	4.7	NO	119	2.95	NA	200	NA	NA	43	92	79
Prophylaxis	M 10.1 y 35 kg	Aplastic anemia	200 mg IV	NA	CsA ^g^	4	2.51	NO	183	2.93	Decreased, 2 times/increased 1 time	100	NA	NA	19	54	51
Prophylaxis	F 14.5 y 61 kg	Aplastic anemia	200 mg Oral	NA	CsA ^g^	2	3.35	NO	109	3.29	NA	200	NA	NA	53	114	40
Prophylaxis	M 8.1 y 25 kg	B-ALL	100 mg Oral	NA	CsA ^g^	3	3.51	NO	15	2.81	NA	100	NA	NA	20	12	15

^a^: Mucormycosis, ^b^: Aspergillosis; F: Female; M: Male; ^c^: Anaplastic Large Cell Lymphoma; ^d^: Myelodysplastic Syndrome; ^e^: Liposomal Amphotericin B; ^f^: Caspofungin; ^g^: Cyclosporine; ^h^: Serum Creatinine; NA: Not Applicable.

**Table 2:** Review of literature data.

Authors and Date of Publication	No ^a^ of Patients	No ^a^ of Patients <30 kg	Age (Median y ^b^), Weight (Median kg)	ISA Dose Patients <30 kg	ISA Dose Patients >30 kg	Median ISA C_trough_ (mg/L)	Median ISA C_trough_ (mg/L) Patients < 30 Kg	Median ISA C_trough_ (mg/L) Patients >30 Kg	Total Days of ISA (Range)	IFI Response at Day 90	No ^a^ of Patients with AST >3 Times Upper Limit	No ^a^ of Patients with ALT >3 Times Upper Limit	No ^a^ of Patients with Scr ^c^ >1.5 Times Baseline
Decembrino et al. 2020 [6]	29	7	14.5 y, 47 Kg	100 mg/day	200 mg/day	4.91 (2.15–8.54)	1.1 (0.73–2.15)	5.0 (2.48–8.54)	6–523	12/29 complete	2/29	NA	3/29 spike
Ross et al. 2020 [11]	18	4	12.5 y, 50.2 kg	100 mg/day	200 mg/day	3.6 (0.4–7.4)	3.7 (3.2–5.6)	2.7 (0.4–7.4)	55	6/18 complete	4/18	1/18	2/18
Arrieta et al. 2021 [10]	46	-	11 y, 38 Kg	200 mg/day	200 mg/day	NA	NA	NA	1–26	NA	NA	NA	NA
Ashkenazi-Hoffnung et al. 2020 [7]	4	1	10.5 y 40 kg	5.4 mg/kg/day	200 mg/day	NA	NA	NA	60–115	4/4 complete	NA	NA	NA
Barg et al. 2018 [8]	3	2	5 y, 18.5 kg	100–200 mg/day	200 mg/day	2.32	2.32 (0.98–6.04)	NA	42–236	3/3 complete	NA	NA	NA

^a^: Number; ^b^: years; ^c^: serum creatinine.

## Data Availability

Data is contained within the article.
